# What is the Influence of Prenatal Physical Activity on Body Weight, BMI, and Health Behavior in Seven‐Year‐Old Children?

**DOI:** 10.1155/tsm2/3876111

**Published:** 2026-05-28

**Authors:** Lene Annette Hagen Haakstad, Iselin Kissel, Emilie Mass Dalhaug

**Affiliations:** ^1^ Department of Sports Medicine, Norwegian School of Sports Sciences, Oslo, Norway, nih.no

**Keywords:** exercise, long-term effect, offspring BMI, offspring physical activity, pregnancy

## Abstract

**Objectives:**

To investigate the effects of a prenatal exercise intervention on offspring body weight, body mass index (BMI), and lifestyle variables at 7 years of age.

**Methods:**

Out of 105 physically inactive participants initially randomized to either a physical activity intervention or control group, 80 women and their children (76.2%) participated in this follow‐up study. The intervention consisted of intensive physical activity counseling and supervised, group‐based moderate‐intensity exercise sessions during the second and third trimesters of pregnancy, while the control group received standard prenatal care only. Data were collected via a standardized telephone interview with the mother and included the offspring’s body weight and height, physical activity level, sports participation, and adherence to general health recommendations regarding screen time and diet. Differences between groups were examined using two‐sided independent sample *t*‐tests or chi‐squared tests as appropriate.

**Results:**

Mean BMI was similar in both groups (intervention group: *n* = 40; control group: *n* = 40), but the intervention group had fewer children with overweight/obesity (1 vs. 4) and underweight (3 vs. 6) than the control group. There was a nonsignificant trend toward increased physical activity among the offspring of the intervention group compared with the control group. This was reflected in a higher number following guidelines (≥ 60 min of daily moderate‐to‐vigorous physical activity [MVPA] according to WHO recommendations) (97.5% vs. 87.5%), number of bouts of MVPA per week (6.4 ± 3.8 vs. 5.8 ± 2.3), and daily hours of MVPA in both summer and winter seasons (2.4 ± 1.3 vs. 2.2 ± 1.3 and 1.6 ± 1.0 vs. 1.4 ± 1.0). The intervention group also reported higher rates of active school transportation than the control group (72.5% vs. 57.5%). No differences were observed in sedentary screen time and diet between the groups.

**Conclusions:**

Our results suggest that the intervention may have positively influenced the BMI status and physical activity levels of the offspring.

## 1. Introduction

Pregnancy may represent an important period for influencing child health, as maternal behaviors during gestation may affect the intrauterine environment and potentially shape future health outcomes in the offspring. Women of reproductive age are particularly susceptible to weight gain, and pregnancy is often accompanied by reductions in physical activity, which may lead to excessive gestational weight gain (GWG) [1, 2]. While these changes have clear implications for maternal health, they may also have consequences for the developing fetus and later child health. A higher maternal body mass index (BMI) and an inactive lifestyle during pregnancy have been associated with increased risks of offsprings with overweight and obesity, conditions that are linked to long‐term health challenges [3, 4]. Thus, preventive strategies during pregnancy that promote appropriate GWG and regular physical activity may be relevant not only for maternal health but also for the health of the next generation.

Pregnancy may also be a suitable period for behavioral change, as expectant mothers tend to be more receptive to health information and lifestyle advice [5–7]. Engagement in physical activity interventions during pregnancy may increase the likelihood that healthy behaviors are maintained after childbirth, potentially influencing the family environment in which the child grows up [8, 9].

The Barker hypothesis suggests that the intrauterine environment of a developing fetus may influence future health and disease risk. Unhealthy maternal behaviors and exposures during pregnancy may increase these risks, whereas healthy behaviors such as appropriate GWG and physical activity may have a more favorable influence on later offspring health outcomes [10]. Epidemiological studies support the notion that in utero conditions may have lasting effects on offspring health [9, 11]. However, parental lifestyle behaviors after pregnancy may also influence children’s health outcomes, as parents act as important role models, making causal interpretation challenging [12]. Therefore, randomized controlled trials (RCTs) are needed to better establish whether prenatal lifestyle interventions may have long‐term effects on child health.

The pregnancy exercise intervention was an RCT designed to examine the effects of a supervised, twice‐weekly aerobic dance program combined with intensive physical activity counseling on GWG in physically inactive nulliparous women. Previous analyses showed that regular adherence to the intervention resulted in significantly lower GWG compared with the control group, and no participants exceeded the Institute of Medicine (IOM) recommendations [13]. Furthermore, women in the intervention group demonstrated lower body weight and BMI, as well as higher levels of physical activity at the 7‐year follow‐up [8].

To date, few studies have investigated the long‐term effects of prenatal lifestyle or exercise interventions on offspring health outcomes beyond preschool age, including weight status, BMI, and health behavior [12, 14, 15]. Moreover, only a limited number of RCTs have included supervised exercise delivered by health professionals [14, 16]. Therefore, the aim of the present study was to investigate the effects of an exercise and physical activity intervention during pregnancy on offspring body weight, BMI, and health behavior at 7 years of age. We hypothesized that the intervention may have a small positive influence on the child’s health behavior.

## 2. Materials and Methods

The present follow‐up study and trial modifications were conducted following the Helsinki Declaration and received ethical approval from the Regional Committee for Research Ethics in Medical and Health Research, South‐East Norway (2014/2034/REK, 09/03/2015). The trial is registered with the ID NCT006171149 at ClinicalTrials.gov (last update posted September 18, 2009).

Between September 2007 and November 2008, we conducted the pregnancy exercise intervention trial in Oslo, Norway. Nulliparous women were recruited from antenatal clinics if they did not engage in a structured exercise program involving ≥ 60 min of moderate‐to‐vigorous physical activity (MVPA) per week. Women were randomized between gestational Weeks 12 and 24 to an intervention group or a control group, and the intervention continued until late pregnancy (gestational Weeks 36–38), resulting in a variable total intervention duration depending on gestational age at inclusion. Participants were recruited individually and were not clustered within specific schools or neighborhoods. Exclusion criteria were based on the American College of Obstetricians and Gynecologists (ACOG) guidelines at the time [17], which included severe heart disease, poorly controlled thyroid disease, pregestational diabetes or gestational diabetes mellitus, pre‐eclampsia, pregnancy‐induced hypertension, a history of more than two miscarriages, and persistent bleeding after Week 12 of gestation, as well as other conditions that could interfere with participation. Moreover, women who could not attend at least two exercise classes per week were not eligible. At trial inclusion, no notable differences in demographic characteristics were observed between the intervention and control groups [13]. Educational level and occupation were collected at baseline and are reported in the original RCT; these variables are therefore not repeated in the present long‐term follow‐up. No additional socioeconomic variables (e.g., income) were collected.

**FIGURE 1:**
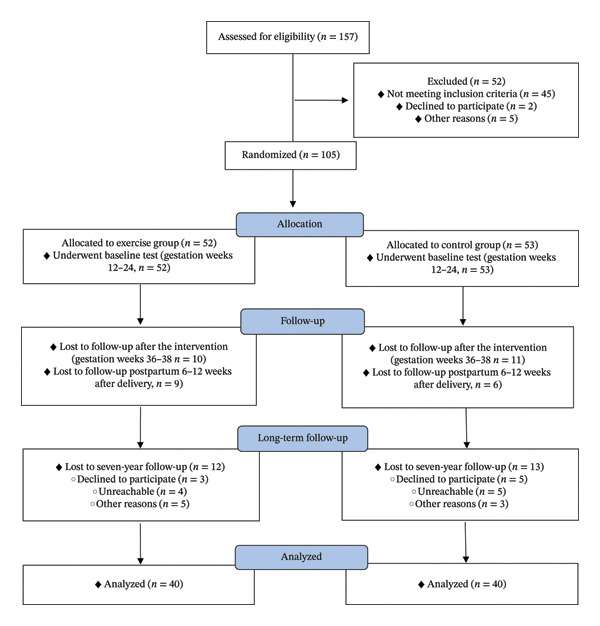
Flowchart of the participants in relation to the long‐term analysis of child health outcomes.

This follow‐up study involved a 50‐min standardized telephone interview with the mothers, conducted seven years after the initial trial (March 2015 to October 2015) [8]. The interviews were conducted continuously during this period and were not scheduled according to group allocation. All the interviews were done by two investigators who were blinded to group allocation during data collection and asked questions in a consistent manner and order. Of the participants originally randomized, 80 out of 105 (76.2%) provided written consent to take part in the follow‐up. Five participants completed the protocol and questions via email due to their inability to participate in the telephone interview. Figure 1 displays a CONSORT diagram that provides a comprehensive flowchart of the participants in relation to the long‐term analysis of child health outcomes.

### 2.1. Intervention and Control Group

In the original trial, the intervention group received intensive counseling to encourage them to engage in moderate physical activity for at least 30 min on most days of the week, following ACOG guidelines [17]. Additionally, they were given access to supervised group exercise sessions tailored for pregnant women [17]. The sessions typically included a 10‐min warm‐up, 35 min of moderate‐intensity cardiovascular exercise, and 15 min of strength training focusing on the back muscles, deep abdominal stabilization muscles, and pelvic floor muscles. To accommodate participants′ full‐time work schedules, the exercise groups were held in the evenings for 60 min three times per week. For further details on the physical activity counseling program and group exercise, please see Haakstad and Bo [18]. The participants were not given any financial compensation, but all exercise sessions and physical activity counseling were provided free of cost.

Both the intervention group and the control group received routine prenatal care following Norwegian standards, which included nine prenatal consultations and one ultrasound examination in the second trimester [19]. Prenatal care in Norway is provided by midwives and physicians free of charge.

### 2.2. Outcome Measures

Primary outcomes for the study were focused on three areas: children’s BMI status, level of physical activity, and sport participation. Secondary outcomes included adherence to general health recommendations related to screen time and diet. The standardized interview guide was based on previous questions used in the Norwegian Mother and Child Cohort Study (MoBa) [20].

#### 2.2.1. BMI Status

Mothers provided information on their offspring’s weight (kg) and height (m), which was then used to calculate BMI (weight (kg)/(height (m) ∗ height (m)). The Norwegian national growth chart for children and adolescents, which incorporates sex‐specific growth patterns, was used to determine the BMI percentile and corresponding classification [21].

#### 2.2.2. Physical Activity and Sport

There were eight questions regarding the children’s physical activity levels, which encompassed the mother’s perception of the number of bouts of MVPA per week and whether the children met the current physical activity recommendations from the World Health Organization (WHO), including a minimum of 60 min of MVPA daily [22]. The duration of physical activity and sports participation was expressed in hours and minutes per day. As commuting to and from school is shown to be a promising strategy to increase daily physical activity [23, 24], we also compared differences between the two groups in active transportation (walking or cycling). Although a compendium of energy expenditures is available to score physical activity questionnaires and estimate children’s energy expenditure levels [25], the standardized questions utilized in the present study needed to be formulated differently to make use of this compendium. Thus, the answers to the questions were used solely to compare the two groups. Finally, by using a Likert scale ranging from 0 to 10, the child’s attitudes toward physical activity, as well as sport/exercise enjoyment, were also reported by the mother. A score of 0 indicated a *strong negative response*, while a score of 10 indicated a *highly positive response*.

#### 2.2.3. Screen Time and Diet

As smartphones, tablets, and widespread internet access have become more prevalent, Norwegian children’s daily sedentary time has increased from 2005 to 2018 [26]. Hence, in this study, we also aimed to compare maternal perceptions of their child’s sedentary behavior in the intervention and control groups by asking the following question: *“How many hours per day does your child typically spend using screens, which includes activities such as watching TV, using a computer, playing video games, using tablets, or using smartphones?”* The question was separated into weekdays and weekends. In addition, five questions were asked about the child’s adherence to dietary recommendations [27]. We first asked the mother to rate the child’s diet on a scale of 0–10 (0 = *very poor*, 10 = *excellent*) based on recommendations for a varied diet including fruits, vegetables, and regular consumption of fish. The second question estimated the child’s daily fruit and vegetable intake, aiming for at least five servings per day, with a numerical response. Finally, the mother responded to how often in a typical week the child (1) ate fish (seafood); (2) consumed sweet beverages (such as soft drinks, juice, or energy drinks); and (3) had snacks (such as potato chips, chocolate, candy, cakes, or ice cream). Answers were given as numbers per week, with the opportunity to select never.

### 2.3. Statistical Analyses

All statistics were conducted with IBM SPSS Statistics 28.0. Distributions of continuous variables were assessed using descriptive statistics and visual inspection of histograms to evaluate approximate normality. Background variables and descriptive statistics are presented as means with standard deviation (SD) or numbers and percentages (%). The principal analysis included 80 participants (intervention group, *n* = 40, and control group, *n* = 40) who completed the 7‐year follow‐up telephone interview. All analyses were conducted according to original group allocation, regardless of exercise attendance. We additionally performed a predefined subgroup analysis restricted to women with high adherence (≥ 24 sessions). In the intervention group, the child’s weight and height were missing for eight and two participants, respectively. In the control group, the corresponding numbers were seven and six, respectively. Differences between the intervention and control groups were examined using independent sample *t*‐tests for continuous variables and chi‐squared tests for categorical variables. Spearman’s rho correlation coefficient was used for ordinal scale variables. Given the limited sample size and the exploratory nature of the 7‐year follow‐up analyses, statistical comparisons were restricted to unadjusted group‐based tests rather than regression‐based models for count or binary outcomes. It should be noted that due to the small differences in the proportion of children with overweight/obesity and underweight in the two groups, it was not feasible to conduct any statistically significant testing. Therefore, these results are presented descriptively without any statistical inferences. No a priori power calculation was performed for the 7‐year follow‐up outcomes, as the sample size was determined by the number of participants available from the original RCT. Due to very low cell counts in BMI categories, analyses of underweight and overweight/obesity were descriptive only, as exact tests or confidence intervals would yield unstable estimates. Given the exploratory nature of the follow‐up analyses and the limited sample size, no formal adjustment for multiple comparisons was applied, and results were interpreted cautiously.

## 3. Results

At the 7‐year follow‐up, a small proportion of the original participants did not accept or could not participate in the interviews. Specifically, this included 23.1% (12/52) of the intervention group and 24.5% (13/53) of the control group. Offspring sex/gender was equally distributed in both groups, and the mean age of the children and their mothers was 7.0 ± 0.23 and 38.2 ± 3.9 years, with no difference between the two groups. Most children had either one or two younger siblings (86.3%). In addition, within the control group, two women were in their third pregnancy during the interview. Nearly all the participants were still living with the father of their firstborn child (90%).

Of the participants originally randomized to the intervention group, prenatal exercise attendance varied widely, spanning from 1 to 55, with a mean of 17.0 ± 12.5 sessions. Fourteen demonstrated high adherence by completing at least 24 exercise sessions during the 2^nd^ and 3^rd^ trimesters.

### 3.1. Long‐Term Effect of the Intervention on the Offspring

#### 3.1.1. BMI (kg/m^2^), Physical Activity Level, and Sports Participation

In the primary analysis, there were no differences in body weight (24.6 ± 3.5 kg vs. 26.0 ± 5.3, *p* = 0.17), height (1.26 ± 5.2 vs. 1.24 ± 22.7, *p* = 0.98), or BMI (15.3 ± 1.3 vs. 15.5 ± 2.2, *p* = 0.65) between the children of the intervention and control groups. However, in a subgroup analysis, which focused on mothers with a high level of adherence to the original intervention (≥ 24 prenatal exercise classes; *n* = 14), a minor effect in the children’s BMI was observed, although it did not reach statistical significance (14.4 ± 1.2 vs. 15.5 ± 2.2, *p* = 0.08). In the entire participant group (intervention, *n* = 40; control, *n* = 40), a total of four offspring from the control group were classified as overweight or obese compared with one in the intervention group. Similarly, the underweight group counted six children from the controls and three from the intervention group.

In the intervention group, 97.5% of mothers perceived that their child met current physical activity guidelines, compared with 87.5% in the control group (*p* = 0.09). Active transportation to school, where the child walks or bikes, was reported by 72.5% of mothers in the intervention group and 57.5% in the control group (*p* = 0.16). The results remained consistent when we conducted stratified analyses based on offspring sex. Table 1 summarizes the offspring’s level of physical activity, including weekly frequency and daily hours of MVPA, with data divided into summer and winter seasons.

**TABLE 1:** Offspring’s level of physical activity, including weekly frequency and daily hours of MVPA^∗^, along with attitudes toward physical activity and sport/exercise enjoyment.

	Intervention group (*n* = 40)	Control group (*n* = 40)	*p*‐value
n (%)			
Following PA guidelines^∗∗^	39 (97.5)	35 (87.5)	0.09
Active school transportation	29 (72.5)	23 (57.5)	0.16
Mean (SD)			
Frequency of MVPA weekly	6.4 (±3.8)	5.8 (±2.3)	0.40
MVPA summer (hr/day)	2.4 (±1.3)	2.2 (±1.3)	0.49
MVPA winter (hr/day)	1.6 (±1.0)	1.4 (±1.0)	0.37
Playing outside summer (hr/day)	3.8 (±1.1)	3.4 (±1.2)	0.12
Playing outside winter (hr/day)	2.2 (±1.1)	1.8 (±1.0)	0.09
Positive toward PA^∗∗∗^	7.7 (±2.1)	7.5 (±2.3)	0.69
Enjoys PA^∗∗∗^	8.4 (±1.5)	7.8 (±2.4)	0.18

^∗^MVPA: moderate‐to‐vigorous physical activity.

^∗∗^The mother’s perception of whether the child met recommendations for daily physical activity.

^∗∗∗^Measured on a 0–10 Likert scale, where 0 represented a *strong negative* and 10 a *highly positive* response.

Table 2 presents offspring participation in organized sports and recreational physical activities based on parental report, with percentages calculated within each group. Group activities on a playground or soccer field were reported as the most common activity/sport, followed by bicycling and swimming.

**TABLE 2:** Offspring participation in organized sports and recreational physical activities (parental report).

Type of sport/activity	Intervention group (*n* = 40)	Control group (*n* = 40)
	*n* (%)^∗^	*n* (%)^∗^
Playing on a playground or soccer field	30 (75%)	30 (75%)
Soccer	16 (40%)	20 (50%)
Bicycling	15 (37.5%)	12 (30%)
Swimming	14 (35%)	11 (27.5%)
Gymnastics	9 (22.5%)	5 (12.5%)
Handball	4 (10%)	8 (20%)
Dancing	7 (17.5%)	5 (12.5%)
Cross‐country skiing	3 (7.5%)	6 (15%)
Running/orientation	1 (2.5%)	2 (5%)
Martial arts	1 (2.5%)	1 (2.5%)
Ice hockey	0	1 (2.5%)
Other activities	9 (22.5%)	10 (25%)

^∗^Percentages do not total 100%, as participants were given the option to select up to three responses.

#### 3.1.2. Screen Time and Diet

Overall, the offspring’s screen time and dietary habits in the intervention group did not show significant differences from those of the control group (Table 3).

**TABLE 3:** Offspring’s screen time and diet.

	Intervention group (*n* = 40)	Control group (*n* = 40)	*p*‐value
Mean (SD)			
Sedentary time^∗^			
Weekday (hr/day)	0.9 (±0.4)	1.0 (±0.4)	0.27
Weekend (hr/day)	2.6 (±0.8)	2.4 (±0.8)	0.27
Compliance with dietary guidelines^∗∗^	7.3 (±1.9)	7.2 (±2.0)	0.82
Fruit and vegetable intake (daily)	3.6 (±1.5)	3.7 (±1.3)	0.75
Fish intake (weekly)	2.6 (±1.5)	2.8 (±1.9)	0.60
Drinking sweet beverages (weekly)	2.6 (±2.8)	3.0 (±2.9)	0.53
Snack consumption (weekly)	1.8 (±0.8)	2.1 (±0.8)	0.09

^∗^Including activities such as watching TV, using a computer, tablet, smartphone or playing video games.

^∗∗^Measured on 0–10 Likert scale, where 0 represented *not at all* and 10 *very well*.

## 4. Discussion

At a 7‐year follow‐up, our primary analysis showed no differences in children’s mean BMI between the intervention and control groups. However, in a subgroup analysis with mothers showing high adherence to the exercise intervention, a minor effect on children’s BMI was observed. It is worth noting that the intervention group had fewer children with overweight/obesity (1 vs. 4) and underweight (3 vs. 6) than the control group. Moreover, in all measurements of physical activity, children from the intervention group consistently exhibited higher scores/numbers than those in the control group, although these observations did not reach statistical significance. Screen time and dietary habits showed no differences between the groups.

Pregnancy offers a unique opportunity to influence the intrauterine environment and enhance the child’s future health through maternal behavior adjustments like exercise [9–11]. However, there are few RCTs on long‐term effects on offspring body weight, BMI, and health behavior following exercise interventions during pregnancy (Chiavaroli et al., 2018 [12]).

The pregnancy exercise intervention was completed as a public health approach, evaluating the effects of a supervised, twice‐weekly aerobic dance program and intensive physical activity counseling on GWG in inactive nulliparous women. We have previously reported that participants adhering to regular exercise significantly reduced maternal weight gain compared with the control group, and none exceeded the IOM weight gain recommendations [13]. A growing body of evidence also suggests that adopting a healthy lifestyle with prenatal exercise could benefit not only the mother but also potentially prevent the inheritance of overweight/obesity through developmental programming of BMI status [28–30]. For instance, studies examining the placenta in human subjects have demonstrated that pregnant individuals who exercise tend to exhibit greater placental growth and enhanced functionality when compared to their sedentary counterparts [29, 31]. As a result, maternal exercise has the potential to influence the in utero environment, enhancing fetal‐placental development and health by regulating placental blood flow, nutrient distribution, hormones, and oxygen transport [29, 32].

While our study did not yield statistically significant results in the primary analysis, the insights gained from the subgroup analysis and the disparities in childhood weight categories between groups suggest that prenatal exercise done on a regular basis may have subtle impacts on offspring’s BMI and weight status. However, given the limited sample size, the results must be viewed with caution, and we need further investigation with a larger sample size to solidify this trend.

In line with our main findings, two systematic reviews and one meta‐analysis of randomized trials [12, 33, 34] failed to establish any significant associations between prenatal exercise and children with overweight/obesity and adiposity (skinfold thickness, body weight, and BMI) in the follow‐up studies. Our data are also supported by a Norwegian RCT follow‐up study, showing that randomization to prenatal exercise did not affect offspring BMI at 7 years of age [14]. Similar to our follow‐up study, the participants in this RCT consisted of normal‐weight, healthy pregnant women [14]. It is worth adding to the discussion that prenatal lifestyle interventions targeting those with maternal obesity and excessive weight gain have shown some success in reducing infant obesity up to 12 months of age [35, 36]. This is strengthened by a large prospective cohort study of pregnant women and their children, which found that mothers with excessive GWG had children with a higher risk of being overweight in early childhood [37]. Also, animal studies, specifically in mice and rats, lend additional support to this, showing that the impact of maternal exercise in increasing lean muscle mass and decreasing fat mass was most prominent in the offspring of mothers who were obese [29].

According to WHO’s guidelines, children and adolescents (5–17 years) should aim for at least 60 min of MVPA per day, including a variety of aerobic activities (such as running, swimming, and dancing) and muscle‐strengthening activities (such as climbing, playing, and sport participation) [22]. This recommendation aligns with guidelines from Norway [38] and is consistent with WHO’s previous guidelines from 2010 [39]. Baran et al. [40] found that not following WHO’s recommendations increased the risk of overweight and obesity in children and adolescents, and more than 60 min of daily MVPA was associated with a healthier body weight. In our study, 97.5% of mothers in the intervention group and 87.5% in the control group believed their child met the current physical activity guidelines at the 7‐year follow‐up, with no sex differences. These proportions align with device‐measured physical activity in six‐year‐old Norwegian children, which showed that 94% of boys and 87% of girls met the recommendations [41].

Engaging in a prenatal lifestyle and physical activity intervention may promote the continuation of these practices postchildbirth [8, 9], and parents serve as influential role models in their children’s lives [42]. Children from the intervention group consistently showed higher physical activity scores than those in the control group, potentially due to a supportive parental environment encouraging healthier habits. However, without statistical significance, these observations may just be random, due to reporting bias or other unaccounted variables. Further investigation is required to establish causality and understand the underlying factors driving these differences in physical activity scores between the two groups.

In the 7‐year follow‐up study conducted by Bjøntegaard et al. [14], the results showed that boys in the control group spent more time using electronic devices than boys in the intervention group, which contrasts our results. Despite this discrepancy, our research aligns with theirs in key aspects related to the children’s physical activity levels. Additionally, both studies share significant parallels in study design, incorporating similar prenatal exercise interventions and follow‐up data collection timing. These similarities enhance the comparability of our respective findings, providing an interesting backdrop for the screen time differences.

Although lifestyle interventions during pregnancy have demonstrated improvements in maternal dietary behaviors and a modest impact on GWG, there is no evidence of a positive influence on the child’s diet and nutritional habits [34], mirroring the findings from our study. In addition, consistent with other reports [34, 43], most children fell short of the recommended daily fruit and vegetable servings (≥ 5) while exceeding their intake of calorie‐dense snacks like potato chips, chocolate, candy, cakes, and ice cream, as well as sweet beverages. The health benefits of fruit and vegetable consumption from an early age are well recognized, and suboptimal eating habits in childhood are likely to persist into adolescence and adulthood [44, 45].

### 4.1. Methodological Discussion

Despite some loss to follow‐up, previous analyses indicated no significant differences in demographic characteristics between participants and nonparticipants at the 7‐year follow‐up [8]. Moreover, nearly 80% of the original participants, equally distributed in intervention and control groups, were successfully re‐contacted and participated in the present long‐term follow‐up study. In addition, the baseline characteristics of the participants closely align with those of the Norwegian MoBa Study [46], indicating a representative sample and demonstrating the comparability of our results to other pregnant women in Norway. However, the results should be viewed with caution when considering pregnant populations characterized by non‐Scandinavian origin, low educational level, higher BMI, and varying health conditions.

Opting for telephone interviews as opposed to paper or electronic surveys may have minimized the likelihood of misinterpreting questions regarding a child’s anthropometric measurements, physical activity levels, engagement in sports, and adherence to general health recommendations concerning screen time and dietary habits. Furthermore, all the interviews were done by two investigators only, ensuring that questions were asked in the same manner and order, with both blinded to group allocation during data collection. Besides, in an RCT with randomized group allocation, the study design helps reduce the risk of differential reporting bias, including recall and social desirability bias, when relying on maternal reports for assessing children’s health and lifestyle behaviors. The absence of objective measures of physical activity, such as accelerometers, further limits the precision of the physical activity estimates; however, the standardized questionnaire‐based approach allowed for consistent comparisons between the randomized groups.

Although the Norwegian national growth chart, which accounts for sex‐specific growth patterns [47], was used to determine BMI percentiles and classifications, it is important to note that BMI is not a precise measure of children’s body composition. Hence, for more accurate data, it is advisable to gather information from various sources like skinfold thickness measurements and dual‐energy x‐ray absorptiometry (DXA) [12]. While DXA is the most accurate method, it is expensive, time‐consuming, and requires competent staff.

Unfortunately, we do not know whether the mother measured or estimated the child’s height and body weight. Still, it is unlikely to have affected the groups differently. The mean BMI of the seven‐year‐old children in our study was comparable with parent‐reported data in another large cohort study with children in the same age group [48]. Moreover, our interview questions regarding physical activity and sports participation align with established practices in other Norwegian studies [20, 49].

A limitation of this study is that no a priori power calculation was conducted for the 7‐year follow‐up outcomes, including BMI categories and offspring outcomes. The original RCT was powered for GWG during pregnancy, not for long‐term offspring outcomes. Consequently, the present follow‐up analyses should be considered exploratory, and the study may have been underpowered to detect small differences between groups. Second, a small number of participants adhered to the original exercise intervention, diminishing the likelihood of observing meaningful effects. Similar to the “no effect without taking the pill” scenario, in exercise interventions, if participants do not consistently engage in the prescribed exercise program, it becomes difficult to measure the intended benefits. Finally, despite a respectable number of participants attending the follow‐up study, it is important to consider the possibility of being underpowered to detect significant differences in various outcomes. Therefore, results lacking statistical significance should be interpreted with caution.

## 5. Conclusion

While our findings hint at a small favor in BMI status in the intervention group, with fewer children with overweight/obesity and underweight compared with the control group, they should be interpreted cautiously due to the limited sample size and the exploratory nature of the follow‐up. Likewise, tendencies toward higher compliance with activity guidelines, frequency of MVPA, and active school transportation in offspring of the intervention group compared with controls should be interpreted with caution and confirmed in larger‐scale research, including extended follow‐up into adolescence.

**Nomenclature**
ACOG: American College of Obstetricians and Gynecologists
BMI: Body mass index
DXA: Dual‐energy X‐ray absorptiometry
GWG: Gestational weight gain
IOM: Institute of Medicine
MoBa: The Norwegian Mother and Child Cohort Study
MVPA: Moderate‐to‐vigorous physical activity
RCT: Randomized controlled trials
WHO: World Health Organization

## Author Contributions

Lene Annette Hagen Haakstad conceived the idea for the present follow‐up study and wrote the protocol together with Iselin Kissel. Iselin Kissel was responsible for participant follow‐up and data collection. Lene Annette Hagen Haakstad supervised the project and outlined the manuscript together with Emilie Mass Dalhaug.

## Funding

The pregnancy exercise intervention was supported by one PhD position at the Norwegian School of Sport Sciences, Department of Sports Medicine, including the use of their exercise facilities free of charge. The authors did not receive support from any organization for the submitted work.

## Disclosure

All authors read and corrected draft versions of the manuscript and approved the final version. The funders had no role in study design, data collection and analysis, decision to publish, or preparation of the manuscript.

## Consent

Informed consent for publication was provided by the participants.

## Conflicts of Interest

The authors declare no conflicts of interest.

## Data Availability

The data that support the findings of this study are available from the corresponding author upon reasonable request.
